# Responses of soil nematode abundance and food web to cover crops in a kiwifruit orchard

**DOI:** 10.3389/fpls.2023.1173157

**Published:** 2023-08-03

**Authors:** Qing-mei Li, Xiao-Xu Qi, Hai-fang Zhang, Yan-jun Zhang, Hong-mei Liu, Jian-ning Zhao, Dianlin Yang, Hui Wang

**Affiliations:** ^1^ Agro-Environmental Protection Institute, Ministry of Agriculture and Rural Affairs, Tianjin, China; ^2^ Key Laboratory of Original Agro-Environmental Pollution Prevention and Control, Ministry of Agriculture and Rural Affairs, Tianjin, China; ^3^ Tianjin Key Laboratory of Agro-Environment and Agro-Product Safety, Tianjin, China

**Keywords:** cover crop, soil nematode community, nematode ecological indices, nematode metabolic footprints, network analysis, orchard ecosystem

## Abstract

Soil biodiversity plays an important role in both agricultural productivity and ecosystem functions. Cover crop species influence the primary productivity of the ecosystem and basal resources. However, it remains poorly understood how different cover crop treatments influence the community of soil nematodes in an orchard ecosystem. In this study, field experiments were conducted to investigate the effects of cover crop treatments with different species numbers, i.e., no cover crop (CK), two cover crop species (C2), four cover crop species (C4), and eight cover crop species (C8), on weed biomass, together with composition, abundance, and metabolic footprint of soil nematode community in a kiwifruit orchard. As compared to the CK group, the groups of cover crop treatments had lower weed biomass, which decreased with the increase of the cover crop diversity. Moreover, for the abundance of total nematodes, fungivores exhibited higher levels in C4 and C8 treatments than that in CK, bacterivores had a higher abundance in C4 treatment, and plant parasites had a higher abundance in C2 and C8 treatments. Cover crop treatments also changed the structure of nematode community and enhanced the nematode interactions and complexity of nematode community network. In addition, C4 increased the Wasilewska index but decreased the plant–parasite index. The metabolic footprints of fungivores were higher in cover crop treatments compared with CK, and C4 and C8 also increased the functional metabolic footprint of nematode. Soil nematode faunal analysis based on nematode metabolic footprints showed that C8 improved the soil nutrient status and food wed stability. Mantel test and redundancy analysis showed that soil microbial biomass nitrogen and carbon, organic carbon, nitrate nitrogen, moisture content, pH, and cover crop biomass were the main factors that affect soil nematode community. In conclusion, cover crop treatments with four or eight plant species displayed a positive role in weed control, improvement of soil health, and promotion of energy flow in the soil food web through the increase in the metabolic footprints of nematodes in kiwifruit orchard.

## 1 Introduction

Soil nematodes are ubiquitous inhabitants of soil ecosystems (Guan et al., 2018), which could reflect the small changes of soil environment, occupy multiple trophic levels, and play a central role in soil food web (Pen-Mouratov et al., 2003; Yeates, 2003). Soil nematodes participate in organic matter decomposition, nutrient mineralization, energy transmission, and plant growth. The species and function diversity of nematode influence plant diversity and biomass, soil microbial biomass, nitrogen mineralization, and ecosystem succession (De Deyn et al., 2004; Jiang et al., 2017). They have been usually used as indicators for assessing the soil health levels, disturbance degree of soil (Bongers et al., 1997; Bongiorno et al., 2019), and ecosystem succession (Tsiafouli et al., 2017; Song et al., 2020). Nematode community varies with soil management practices (such as soil surface crop cover and fertilization) in agroecosystems.

Cover cropping is considered to be one of the main measures for improving soil health, increasing production and quality, and achieving sustainable management of orchard. There is substantial evidence that the abundance and diversity of soil nematode are influenced by cover crop. For instance, the number of enrichment opportunist bacterial feeding nematodes is significantly greater in cover cropped tomato–corn rotations (DuPont et al., 2009). Incorporating cover crops into a corn–soybean rotation is helpful for the diversity and complexity of free-living nematodes, thus enhancing the cycling of energy and nutrients belowground (Leslie et al., 2017). As compared to monocultures, cover crop diversity can amplify ecosystem benefits through simultaneously functioning to increase biological nitrogen fixation, retain nitrogen of soil, and increase total nitrogen (TN) and carbon of soil (Blesh and Martin, 2018; Norton et al., 2019). Moreover, plant diversity enhances root biomass, which increases root-derived organic inputs and the resource availability for soil nematode communities, thus enhancing soil nematode abundance and diversity (Djigal et al., 2012). Previous studies have shown that a high plant diversity provides multifunctionality and maintains more ecosystem services by producing a variety of substrates and altering habitat conditions (Spedding et al., 2004; Carrera et al., 2007; Isbell et al., 2011), which considerably affects nematode community structure (Spedding et al., 2004; Cesarz et al., 2017) and their functions that are related to nutrient cycling (Delgado-Baquerizo et al., 2016; Zhang et al., 2020).

In China, the orchard area is 11.168 million hectares, and the fruit output is 261 million tons, accounting for 20.0% and 15.7% of the world, respectively, ranking among the top in the world (FAO, 2018). For a long time, the extensive management of the orchard, the low degree of standardization, and the problems of fruit quality and safety have severely restricted the sustainable and healthy development of the fruit industry. Cover crop can bring multiple ecological, production, and economic benefits. To date, the positive significance of cover crop on soil health has been generally recognized by international organization experts. However, whether cover crop diversity can bring multiple potential benefits is still unknown. Therefore, a good understanding of the nematode community driven by cover crop species diversity could help extend the functional connections between the above-ground ecosystem and the soil food web in the orchard ecosystem. Our study focused on the responses of nematodes community to cover crop species diversity in the context of orchard systems, documenting the main factors that influence soil nematode community. By establishing a cover crop species diversity experiment, we compared the influences of four cover crop treatments [two cover crop species (C2), four cover crop species (C4), eight cover crop species (C8), and no cover crops as a control (CK)] on the composition, abundance, metabolic footprint of soil nematode, soil organic carbon (SOC), TN, carbon/nitrogen ratio (C/N), nitrate nitrogen (NO_3_^−^-N), ammonium nitrogen (NH_4_^+^-N), pH, soil moisture content (SMC), microbial biomass carbon (MBC), microbial biomass nitrogen (MBN), plant biomass, and diversity. The aims of this study are as follows: 1) to investigate the responses of weed growth, soil nematode abundance, and food web to different cover crop treatments in a kiwifruit orchard; and 2) to determine how the cover crop diversity and soil environmental factors affect soil nematode community under different cover crop treatments in kiwifruit orchard ecosystem.

## 2 Materials and methods

### 2.1 Site description and experimental design

The study was conducted in a kiwifruit planting base of the Economic Crop Research Institute, Shiyan Academy of Agricultural Sciences, Shiyan city, Hubei Province, China (32°50′N, 110°60′E). The mean annual precipitation was 950 mm, with an average annual temperature of 16°C and a frost-free period of 248 days. Prior to the experiment, the soil had the following chemical properties: soil organic matter content of 6.67 g kg^−1^, TN content of 0.44 g kg^−1^, total phosphorus (P) content of 0.49 g kg^−1^, and pH 8.14.

The experiment was conducted in a kiwifruit (*Actinidia chinensis* Planch.) orchard. Kiwifruit trees were planted during 2015 at a spacing of 5 m × 3 m. In 2016, the cover crop experiment was established as a randomized design with three replications. Cover crop treatments include C2, C4, C8, and CK. These four experimental treatments resulted in 12 plots (2 m × 20 m). Before sowing seeds, all plots were ploughed with a small rotary cultivator. All cover crop seeds were sown to inter-row of the kiwifruit trees by hand. In the CK treatment, the weeds were removed regularly by hand, and the weed residues were removed to leave bare soil in the whole process of the experiments. In addition, field management measures are consistent in all plots. The cover crops were mowed down three or four times a year by a mower, and the residues were left on the ground for natural decomposition. Cover crop species were chosen to represent a diversity of plant families (Poaceae, Fabaceae, and Asteraceae) according to different functional groups, with the characteristics of high above-ground biomass, nitrogen fixation, and honey source plants providing habitat and food for predatory natural enemies, and play an important role in improving soil fertility and reducing pests. In addition, the height of cover crops was different, which could make full use of the resources and space, form different levels of community structure, provide different niche for the arthropod, and improve the biodiversity of the kiwifruit orchard. Cover crops applied in our experiment were screened in before the experiment, and all the cover crops could adapt to the local climatic conditions and grow. The cover crop species and seeding rates were shown in **Table S1**. We used the recommendation according to the seed company. In C2 treatment, the sowing rate of the two cover crops was the recommended sowing rate. In C4 treatment, the sowing rate of the four cover crops was the 60% of recommended sowing rate. In C8 treatment, the sowing rate of the eight cover crops was the 30% of recommended sowing rate.

### 2.2 Soil and plant sampling

Ten 20-cm deep soil cores were sampled randomly in each of the 12 plots using a 3.5-cm-diameter soil probe during November 2019 when kiwifruits were harvested. The 10 soil cores were mixed into one composite sample. After removal of roots and stones, each composite sample was divided into two parts, where the first part was stored at 4°C for determining SMC, NO_3_^−^-N, NH_4_^+^-N, MBC, MBN, and nematodes, and the second part was air-dried and stored at room temperature for determining soil pH, SOC, and TN. The samples of plant were collected on August in 2019. In each of the plots, one square area (1 m × 0.5 m) was sampled randomly. All plant species, including weeds, were clipped at the soil surface separately. All plant samples were oven dried for 72 h at 65°C and weighed. Plant Shannon–Weiner index (*H*) was calculated as −∑(Pi × ln Pi), where Pi is the relative abundance of *i*th species in a sample.

### 2.3 Soil physicochemical parameters

SMC was determined using the gravimetric method (oven dried for 24 h at 105°C and weighed); NO_3_^–^-N, and NH_4_^+^-N content were extracted with CaCl_2_ and measured with a continuous-flow analyzer (Bran Lubbe AA3, Germany); and TN content was determined by acid digestion and measured with a continuous-flow analyzer (Bran Lubbe AA3, Germany) (Bao, 2000). SOC content was determined by the K_2_CrO_7_-H_2_SO_4_ oxidation method (Zheng et al., 2018), and SOC/TN (C/N) ratio was calculated. Soil pH was determined in a mixture of water and soil suspension (2.5:1) with electrode method (Chen et al., 2014). The soil MBC and MBN were determined using the chloroform-fumigation extraction and measured with a total organic carbon (TOC) analyzer (Multi C/N 3000, Analytik Jena, Germany) (Wu et al., 2006).

### 2.4 Soil nematode community

Nematodes were extracted using the shallow dish methodology (Mao et al., 2004). Nematode morphological identification was performed according to Bongers (1988) and Yin (1901). The nematode populations were expressed as the number of nematodes per 100 g of dry soil, with 100 nematodes randomly selected for identification from each sample with a microscope. Nematodes were identified to genus and assigned to four trophic groups: bacterivores (Ba), fungivores (Fu), plant parasites (Pp), and omnivores/predators (Op) (Yeates et al., 1993), and classified along the colonizer-persister gradient (c-p values) according to Bongers and Bongers (1998).

Ecological indices for nematode community were calculated as follows: (1) The maturity index (*MI*) and plant–parasite index (*PPI*) were calculated as ∑v(i) × f(i), where v(i) is the c-p value of free-living (plant–parasite) taxa i and f(i) is the proportion of that taxa from the total number of free-living (plant–parasite) nematodes in a sample according to the c-p 1-5 scale. *MI* was used to assess environmental disturbance, and a larger value reflects stable soil conditions and complex soil food web. *PPI* was used to evaluate the damage level of plant–parasite nematodes to plants (Bongers, 1990). (2) The Wasilewska index (*WI*) was calculated as (Ba + Fu)/Pp, where Ba is the abundance of bacterivorous, Fu is the fungivorous abundance, Pp is and the abundance of plant parasites. *WI* was used to indirectly describe the mineralization pathway of organic matter (Yeates, 2003). (3) The nematode channel ratio (NCR) was used to assess the dominant pathway of soil organic matter decomposition and was calculated as Ba/(Ba + Fu), where Ba is the abundance of bacterivorous and Fu is the fungivorous abundance (Neher, 2001). (4) Enrichment index (*EI*) and structure index (*SI*) were calculated as *EI* = 100 × [e/(e + b)] and *SI* = 100 × [s/(s + b)], respectively, where b is the basal food web component (Ba2% and Fu2%), s is the structure component (Ba3%–Ba5%, Fu3%–Fu5%, Om3%–Om5%, and P2%–P5%), and e is the enrichment component (Ba1% and Fu2%). High *EI* and *SI* indicate an enriched environment and a complex or stable food web, respectively (Ferris et al., 2001; Zhao et al., 2021).

The metabolic footprints of nematodes (NMF) indicating carbon utilization in the soil food web based on nematode biomass were computed for each sample. NMF = ∑*N_t_ ×* (0.1×*W_t_*/*m_t_* + 0.273×*W_t_* ^0.75^), where *N_t_*, *W_t_*, and *m_t_* represent the number, fresh weight (µg), and c-p value of t taxa, respectively. The metabolic footprints of bacterivores, fungivores, plant parasites, and omnivores/predators were abbreviated as BaF, FuF, PpF, and OpF, respectively, and were summed to provide different metrics of ecosystem functions (Ferris, 2010). The enrichment footprint (*Fe*) and structure footprint (*Fs*) were calculated according to the life history strategy of nematode. *Fe* reflects the metabolic footprint of nematodes with low c-p values (1~2) and rapid response to resources. *Fs* reflects the metabolic footprint of nematodes with high c-p values (3~5). According to *Fe* and *Fs*, draw four coordinate points for different treatments in the four quadrants A, B, C, and D, (*SI*, *EI*) is the central point. The coordinate points are as follows: (*SI* − 0.5 × *Fs*/k, *EI*), (*SI* + 0.5 × *Fs*/k, *EI*), (*SI*, *EI* − 0.5 × *Fe*/k), and (*SI*, *EI* + 0.5 × *Fe*/k), where k is the conversion constant. Connect four coordinate points in sequence, and the sum of regions delineated by enriched metabolism and structural metabolism is the functional metabolic footprint (Ferris, 2010). The fresh weight of all nematode is obtained from http://nemaplex.ucdavis.edu/Uppermnus/topmnu.htm.

### 2.5 Statistical analyses

The nematode abundances were LN (*x* + 1)–transformed prior to statistical analysis for the normality of data. We analyzed data by one-way ANOVA, to determine the effects of different cover crop treatments (CK, C2, C4, and C8) on plant biomass and diversity, soil properties, nematode abundance, ecological, and metabolic footprint. Data were tested for normality and homogeneity of variance before conducting ANOVA and were log-transformed if they did not satisfy assumptions of normality and homogeneity. In addition, significant differences were analyzed by Duncan’s new multiple differences test at *P* < 0.05. IBM SPSS 22.0 software was performed for these analyses. Figures were created using Origin 8.5 software.

Principal coordinates analysis (PCoA) was calculated to assess the dissimilarities of nematode community structure among the treatments using R. For the co-occurrence network analysis of nematode communities at the genus level, the relative abundance of genus was used in the analyses. A correlation matrix was analyzed using the “psych” package in the R environment, and the co-occurrence network visualization was achieved *via* Gephi (version 0.9.2). Spearman correlations between genera were performed, and the correlations with a coefficient of more than 0.6 and a *P*-value of less than 0.05 were applied. Nematode community networks were built according to MENAP (http://ieg2.ou.edu/MENA/main.cgi) (Wu et al., 2021). Correlations between different nematode trophic groups and plant and soil environmental variables were performed by Mantel tests. Redundancy analysis (RDA) was performed using Canoco for Windows 5.0 to evaluate the relationship between nematode community and plant and soil environmental factors.

## 3 Results

### 3.1 Effects of cover crop treatments on plant community and soil properties

**Figure 1:**
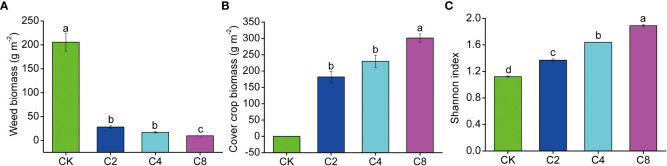
The effects of cover crop treatments on weed biomass **(A)**, cover crop biomass **(B)** and plant community diversity **(C)**. C2, two cover crop species; C4, four cover crop species; C8, eight cover crop species; CK, no cover crop. The different lowercase letter indicates significant differences among treatments according to Duncan's test (P < 0.05). Error bars are standard deviation of means (n = 3).

The plant community composition was reconstructed through cover crops competing with the weeds in the soil seed bank in the orchard ecosystem. Increasing cover crop diversity changed the plant community composition and diversity and effectively controlled the growth of weed. Specifically, compared with CK treatment, cover crop biomass contributed 86.63%~96.89% of the total plant biomass and increased with the increase of cover crop diversity. The proportion of weed biomass covered just 3.11%~13.37% of the total plant biomass and displayed a decreased trend with the increasing of cover crop diversity. In addition, the weed biomass under cover crop diversity treatments was significantly lower than that under CK (F_11_ = 335.300, *P* < 0.001; **Figure 1A**; **Table S2**). Among cover crop treatments, weed biomass under C8 treatment was obviously lower than that under C2 and C4 treatments, whereas cover crop biomass in C8 treatment was significantly higher than that in C2 and C4 treatments (F_11_ = 120.218, *P* < 0.001; **Figure 1B**; **Table S2**). In addition, plant diversity significantly increased with the increase of cover crop species, and C8 > C4 > C2 > CK (F_11_ = 161.153, *P* < 0.001; **Figure 1C**; **Table S2**).

**Figure 2:**
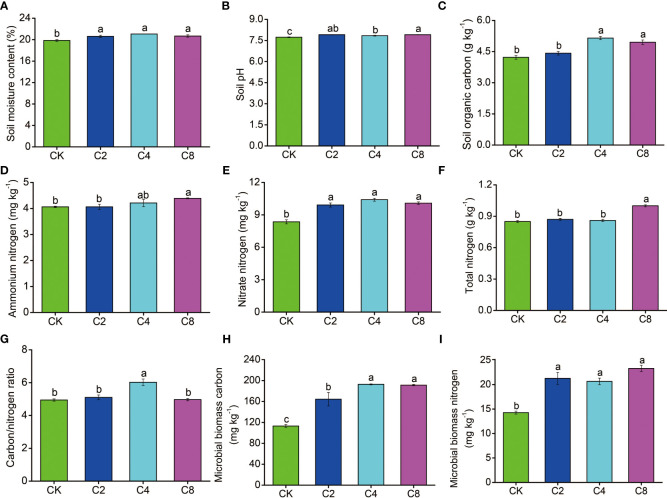
The effects of cover crop treatments on soil moisture content **(A)**, pH **(B)**, organic carbon **(C)**, ammonium nitrogen **(D)**, nitrate nitrogen **(E)**, total nitrogen **(F)**, carbon/nitrogen **(G)**, microbial biomass carbon **(H)** and microbial biomass nitrogen **(I)**. C2, two cover crop species; C4, four cover crop species; C8, eight cover crop species; CK, no cover crop. The different lowercase letter indicates significant differences among treatments according to Duncan's test (P < 0.05). Error bars are standard deviation of means (n = 3).

There were significant differences of cover crop treatments on the soil properties (**Figure 2**). Specifically, compared with CK, SMC (F_11_ = 8.397, *P* = 0.007; **Figure 2A**; **Table S1**), NO_3_^−^-N (F_11_ = 31.833, *P* < 0.001; **Figure 2E**; **Table S2**), MBC (F_11_ = 31.111, *P* < 0.001; **Figure 2H**; **Table S2**), and MBN (F_11_ = 24.764, *P* < 0.001; **Figure 2I**; **Table S2**) significantly increased by 3.83%~6.10%, 18.68%~24.55%, 49.02%~63.24%, and 45.19%~70.32%, respectively, but there were no significant differences among different cover crop treatments, except for the MBC that is higher in C4 and C8 treatments than that in C2 treatment. SOC was also significantly increased in C4 and C8 treatments than that in C2 and CK (F_11_ = 21.947, *P* < 0.001; **Figure 2C**; **Table S2**). In addition, NH_4_^+^-N (F_11_ = 3.109, *P* = 0.089; **Figure 2D**; **Table S2**) and TN (F_11_ = 43.00, *P* < 0.001; **Figure 2F**; **Table S2**) showed significantly higher values in C8 treatment than that in C2 and CK. The pH was higher for cover crop treatments (F_11_ = 15.498, *P* = 0.001; **Figure 2B**; **Table S2**) and increased 0.11~0.18 units, compared with CK; meanwhile, the pH of the C8 treatment was significantly higher than that of the C4 treatment (**Figure 2B**; **Table S2**).

### 3.2 Effects of cover crop treatments on soil nematode community composition and abundance

**Figure 3:**
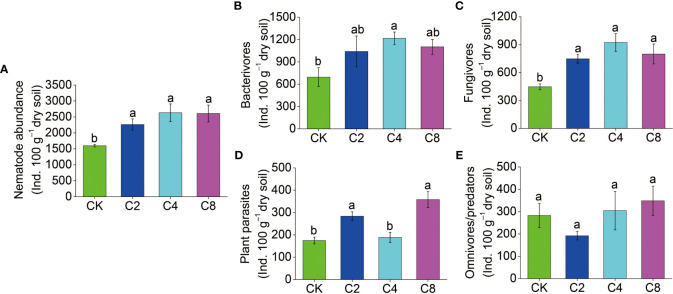
The effects of cover crop treatments on the abundance of soil total nematode **(A)**, bacterivores **(B)**, fungivores **(C)**, plant parasites **(D)** and omnivores/predators **(E)**. C2, two cover crop species; C4, four cover crop species; C8, eight cover crop species; CK, no cover crop. The different lowercase letter indicates significant differences among treatments according to Duncan's test (P < 0.05). Error bars are standard deviation of means (n = 3).

**Figure 4:**
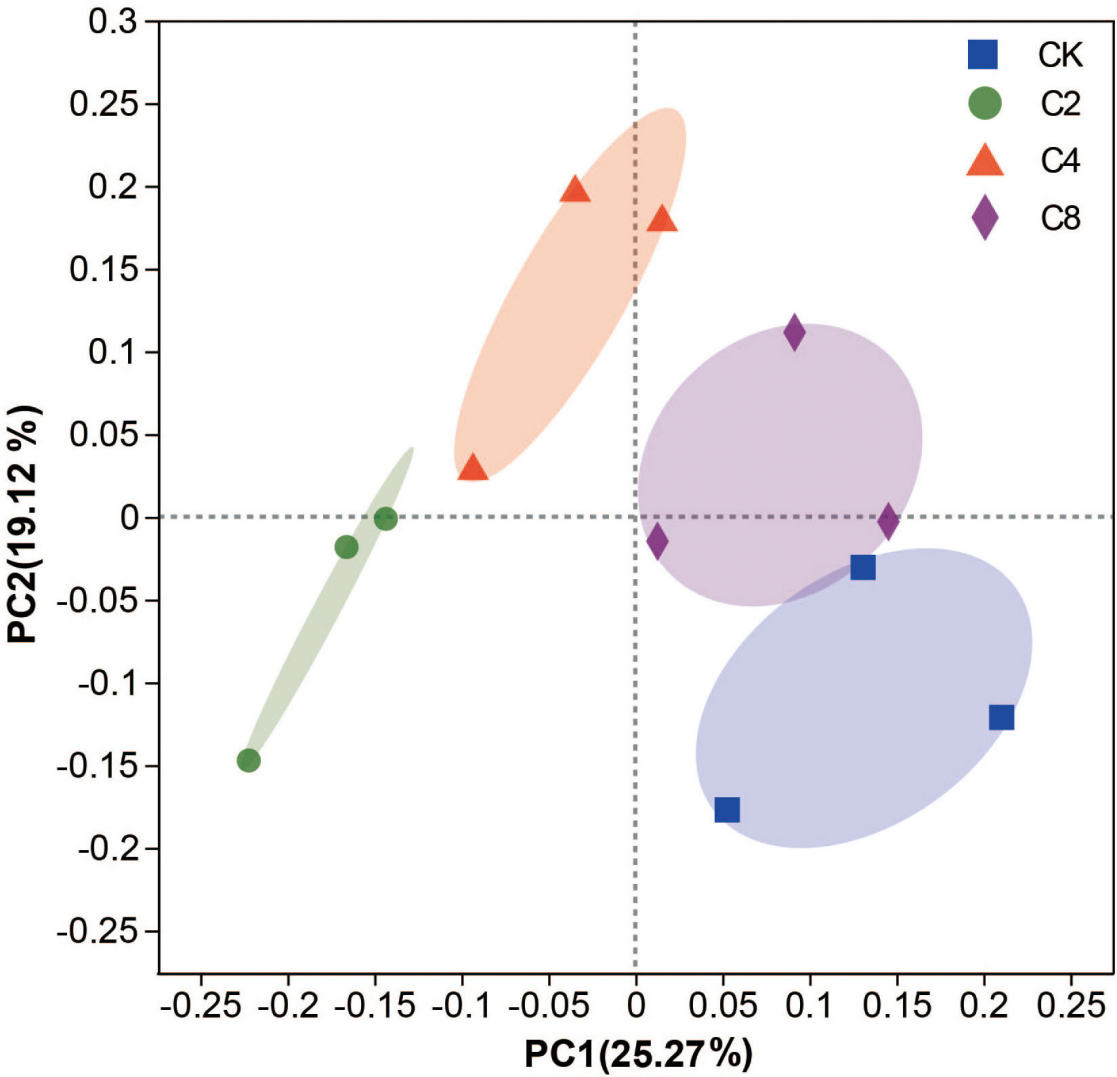
Principal co-ordinates analysis (PCoA) of the nematode community under different cover crop treatments. CK, no cover crop; C2, two cover crop species; C4, four cover crop species; C8, eight cover crop species.

**Figure 5:**
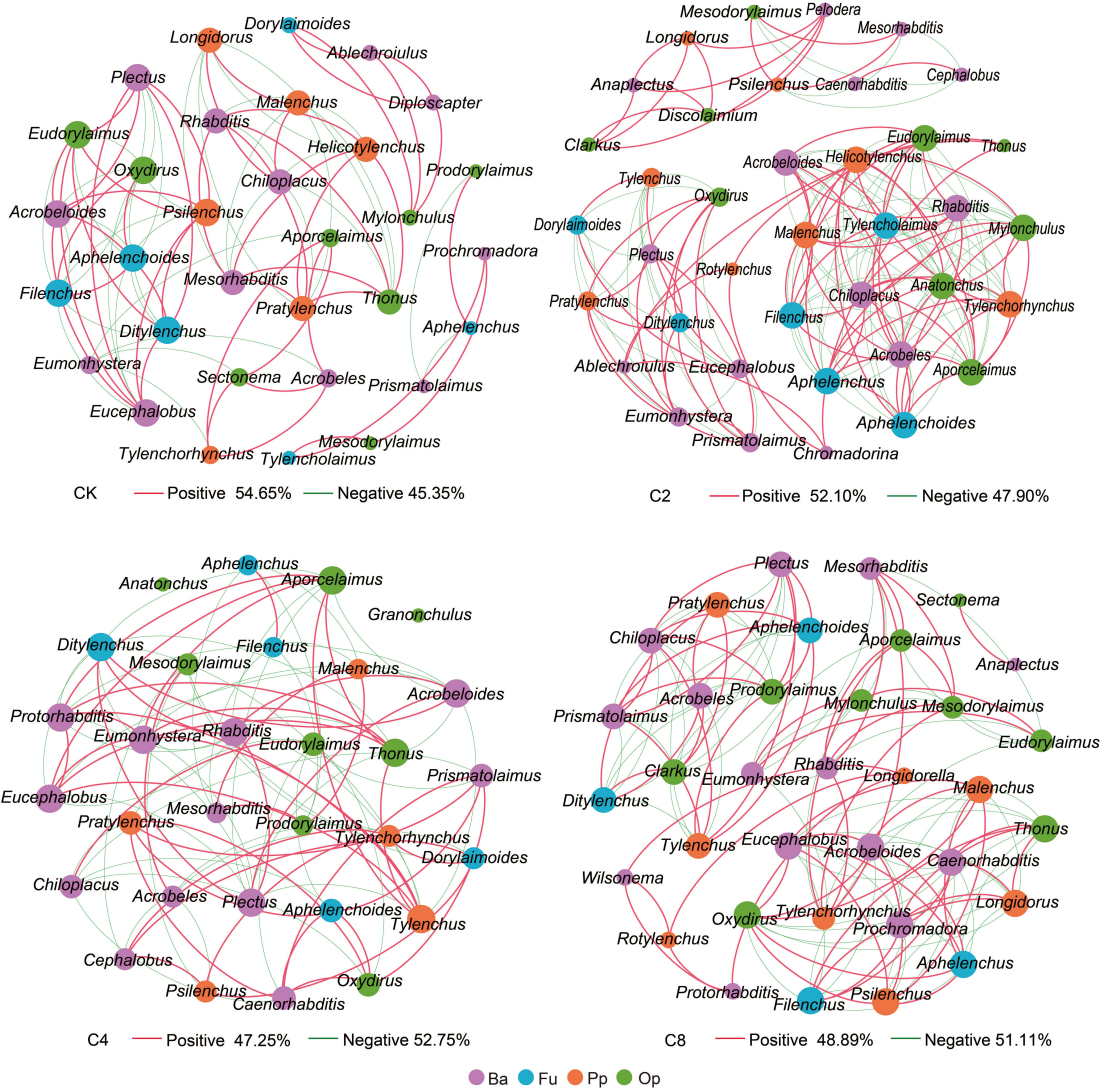
The complexity and interactions of soil nematode community under different cover crop treatments. The connections represented strong (R^2^ > 0.6) and significant (*P* < 0.05) correlations. The size of each node was proportional to the number of connections (degrees). Red lines represented significantly positive and green lines represent significantly negative correlations. Different colors of nodes represented nematode trophic groups. CK, no cover crop; C2, two cover crop species; C4, four cover crop species; C8, eight cover crop species.

Forty-six genera of nematodes were identified in total under different treatments. Bacterivores were found to be the most abundant group with 19 genera, followed by fungivores with 6 genera, as well as omnivores/predators and plant parasites, with 12 and 9 genera, respectively, and *Aphelenchoides*, *Eucephalobus*, and *Rhabditis* were the dominant genera in all treatments (**Table S3**). The total abundance of nematodes increased in cover crop treatments by 41.48%~64.61% and was obviously higher in cover crop treatments compared with that in CK, but no significant differences were observed among different cover crop treatments (F_11_ = 24.119, *P* = 0.009; **Figure 3A**; **Table S2**). Meanwhile, cover crop treatments had effects on different nematode trophic groups. C4 treatment had significantly higher abundance of bacterivores than the CK treatments, but there were no significant differences among different cover crop treatments (F_11_ = 15.116, *P* = 0.121; **Figure 3B**; **Table S2**). Compared with CK, the abundance of fungivores was significantly higher in cover crop treatments, but no significant differences were observed among different cover crop treatments (F_11_ = 31.912, *P* = 0.003; **Figure 3C**; **Table S2**). The abundance of plant parasites was significantly higher in C2 and C8 treatments compared with that in the CK treatment (F_11_ = 5.687, *P* = 0.003; **Figure 3D**; **Table S2**). C4 and C8 treatment slightly increased the abundance of omnivores/predators compared with CK, but there were no significant differences between cover crop and CK treatments (F_11_ = 13.160, *P* = 0.255; **Figure 3E**; **Table S2**). The variations in the structure of soil nematode community from different cover crops treatments were evaluated by PCoA based on nematode relative abundance, and the structures of the nematode communities among CK and cover crop treatments were significantly different (**Figure 4**). The first two principal coordinates for the nematode community represented 25.27% (PC1) and 19.12% (PC2) of total variation in different cover crop treatments. C2 and C4 were significantly different from C8 and CK at the axis 1, and C4 and C8 was significantly separated from C2 and CK at the axis 2. Those results indicated that the nematode community composition was significantly affected by cover crop treatments, and C8 showed a significant difference from C2 and C4 treatments. Network analysis was used to visualize the structural complexity of the soil nematode community, which showed that the networks were complicated by cover crop treatments compared with CK (**Figure 5**). In the networks, most nodes were derived from Ba, which showed increasing node numbers under cover crop treatments except for C4. Total nodes in the network were higher in C2 and C8 treatments, and the connectivity in the cover crop treatments was 5.81%~94.19% higher than that in CK (**Table S4**). Other network parameters, including average degree and network density, were also higher with cover crop treatments (**Table S4**). There were more positive correlations in the nematode networks of CK and C2, whereas C4 and C8 had more negative correlations in the nematode networks. The taxa that controlled the nematode network were also different under cover crop treatments. At the genus level, taxa in Ba (*Eucephalobus*, *Plectus*, and *Acrobeloides*), Fu (*Ditylenchus*, *Aphelenchoides*, and *Filenchus*), Pp (*Psilenchus*), and Op (*Eudorylaimus*, *Oxydirus*) controlled the nematode network in the CK soils. Taxa in Ba (*Chiloplacus*, *Acrobeloides*, *Acrobeles*, and *Rhabditis*), Fu (*Aphelenchus*, *Aphelenchoides*, *Filenchus*, and *Tylencholaimus*), Pp (*Malenchus* and *Helicotylenchus*), and Op (*Mylonchulus*, *Anatonchus*, *Eudorylaimus*, and *Aporcelaimus*) controlled the nematode network in the C2 soils. The taxa that controlled the nematode network in the C4 soils were Ba (*Plectus*, *Acrobeloides*, *Rhabditis*, *Protorhabditis*, *Eucephalobus*, and *Eumonhystera*), Fu (*Ditylenchus*), Pp (*Tylenchus*), and Op (*Thonus* and *Aporcelaimus*). The taxa that controlled the nematode network in the C8 soils were Ba (*Acrobeloides*, *Prochromadora*, *Caenorhabditis*, and *Eucephalobus*), Fu (*Aphelenchus* and *Filenchus*), Pp (*Malenchus* and *Longidorus*), and Op (*Thonus* and *Oxydirus*).

### 3.3 Effects of cover crop treatments on ecological indices and metabolic footprint of nematode

**Figure 6:**
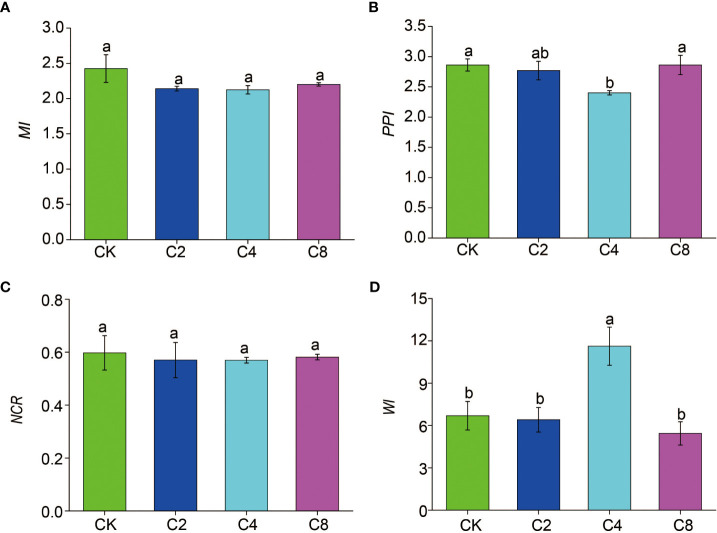
The effects of cover crop treatments on the ecological indices of soil nematode communities including MI **(A)**, PPI **(B)**, NCR **(C)** and WI **(D)**. C2, two cover crop species; C4, four cover crop species; C8, eight cover crop species; CK, no cover crop. MI, maturity index; PPI, plant–parasite index; NCR, nematode channel ratio; WI, Wasilewska index. The different lowercase letter indicates significant differences among treatments according to Duncan's test (P < 0.05). Error bars are standard deviation of means (n = 3).

**Figure 7:**
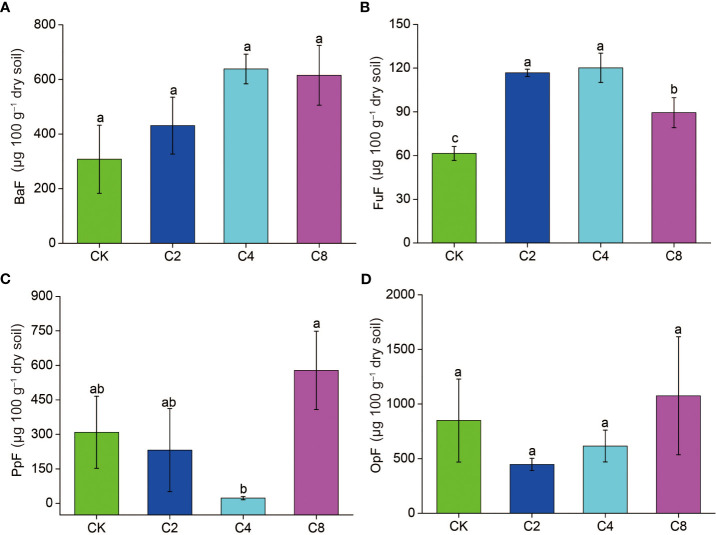
The effects of cover crop treatments on the Metabolic footprint of nematode subjected including BaF **(A)**, FuF **(B)**, PpF **(C)** and OpF **(D)**. C2, two cover crop species; C4, four cover crop species; C8, eight cover crop species; CK, no cover crop. BaF, Bacterivores metabolic footprint; FuF, Fungivores metabolic footprint; PpF, plant parasites metabolic footprint; OpF, Omnivores/predators metabolic footprint. The different lowercase letter indicates significant differences among treatments according to Duncan's test (P < 0.05). Error bars are standard deviation of means (n = 3).

**Figure 8:**
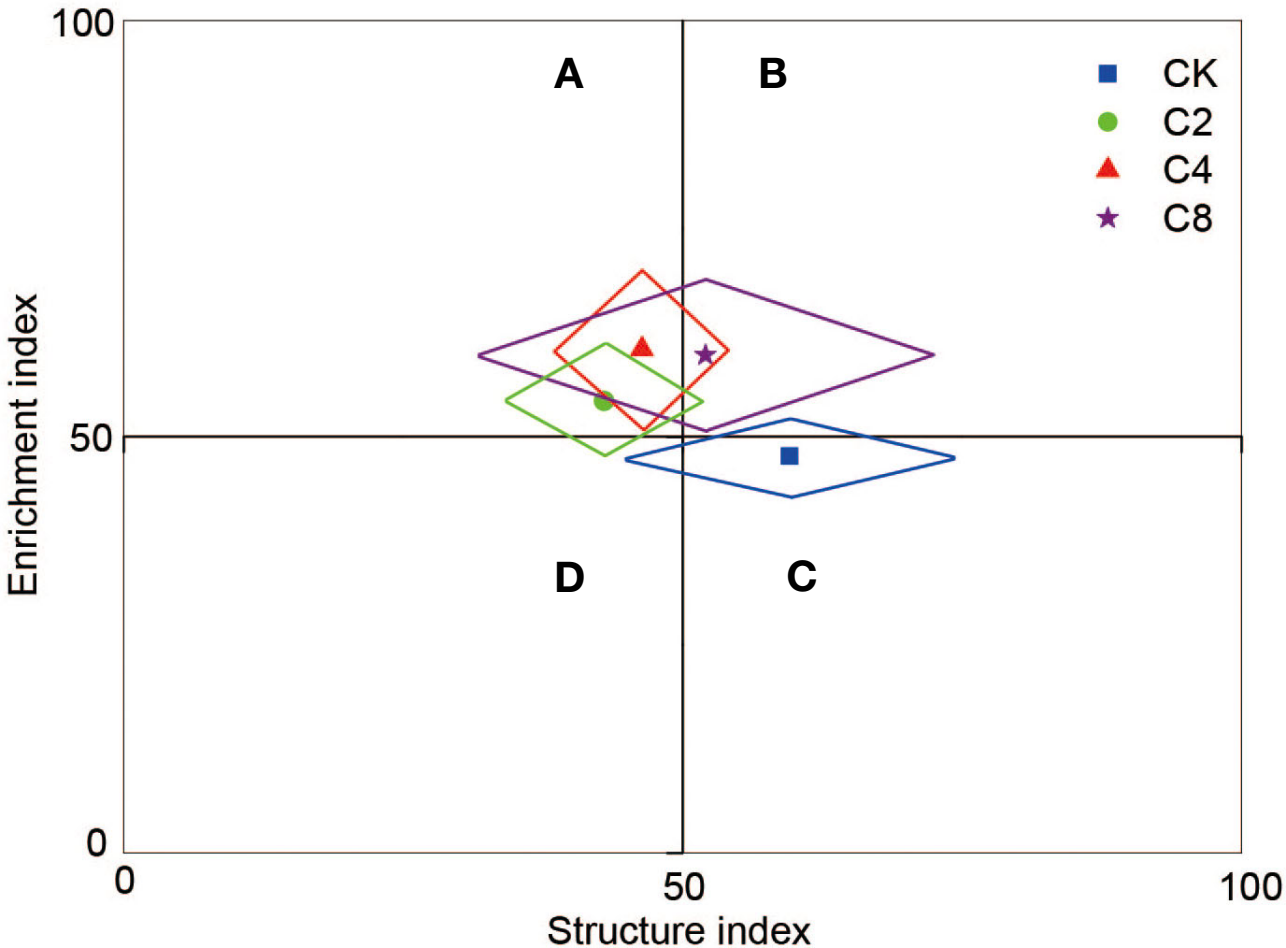
Faunal analysis of soil food web under different cover crop treatments (based on nematode metabolic footprints). C2, two cover crop species; C4, four cover crop species; C8, eight cover crop species; CK, no cover crop. Quadrat A, the soil environment is highly disturbed, and the food web is disturbed to a certain extent; Quadrat B, the soil nutrient status is better and the soil is less disturbed, and the food web is mature and stable; Quadrat C, the soil nutrient enrichment is poor and the disturbance is small, and the soil food web is in a structured state; Quadrat D, the soil nutrient enrichment status is poor and the disturbance degree is high, which has caused stress to the environment and the food web is degraded.

C4 significantly decreased *PPI* (F_11_ = 3.160, *P* = 0.086; **Figure 6B**; **Table S2**) but increased *WI* (F_11_ = 7.203, *P* = 0.012; **Figure 6D**; **Table S2**) compared with CK, C2, and C8 treatments. However, there were no significant differences in *PPI* and *WI* between C2, C8, and CK (**Figures 6B D**). It showed no significant differences in *MI* and *NCR* between cover crop treatments and CK (**Figures 6A, C**). Moreover, cover crop treatments changed the metabolic footprint of soil nematode (**Figure 7**). Cover crop treatments slightly increased the metabolic footprint of bacterivores compared with CK but showed no significant differences compared with CK treatment (**Figure 7A**). Compared with CK, cover crop treatments increased metabolic footprint of fungivores group, and the fungivores metabolic footprint of the C2 and C4 treatments was significantly higher than that of the C8 treatment, but it showed no significant differences in fungivores metabolic footprint between C2 and C4 treatments (F_11_ = 12.693, *P* = 0.002; **Figure 7B**; **Table S2**). Compared with CK, C2 and C4 slightly decreased metabolic footprint of plant parasites and omnivores/predators, whereas C8 slightly increased metabolic footprint of which, but all cover crop treatments showed no significant differences compared with CK treatment (**Figures 7C, D**). The *Fe* was also increased in cover crop treatments and was significant higher in C4 and C8 treatments as compared with that in CK (F_11_ = 3.238, *P* = 0.079; **Table S5**), whereas there was no significant difference in *Fs* between cover crop treatments and CK. Soil nematode faunal analysis based on nematode metabolic footprints showed that the plot of C8 was situated in quadrat B, which suggested that the soil nutrient status is better, the soil is less disturbed, and the food web is mature and stable, whereas those of CK in quadrat C indicated that the soil nutrient enrichment is poor and less disturbed environments (**Figure 8**; **Table S6**). In addition, functional metabolic footprint of nematode in C8 was larger than that in CK, whereas C2 and C4 treatments were situated in quadrat A, which showed that the soil nutrient status is better in C2 and C4 treatments compared with CK (**Figure 8**; **Table S6**).

### 3.4 Relationships between nematode community and plant and soil properties

**Figure 9:**
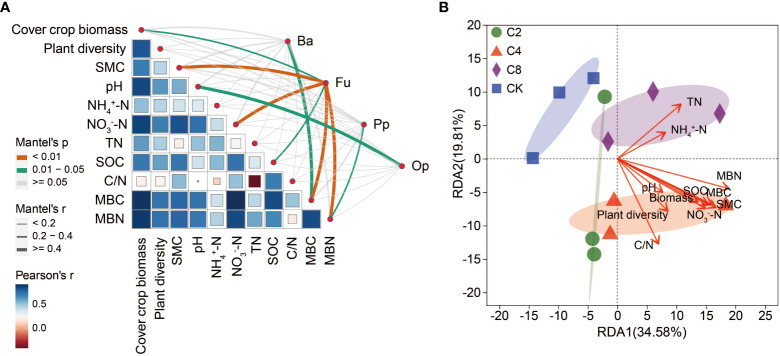
Mantel test **(A)** and redundancy analysis **(B)** between plant, soil physicochemical factors and soil nematode community. SMC, soil moisture content; NO3-N, nitrate nitrogen; TN, total nitrogen; SOC, soil organic carbon; MBC, microbial biomass carbon; MBN, microbial biomass nitrogen.

The Mantel test analysis revealed that soil nematode community was strongly associated with plant and soil environmental factors (**Figure 9A**). The bacterivores community was strongly positively correlated with MBC (Mantel’s r > 0.4, *P* < 0.05). The fungivorous community was strongly positively correlated with cover crop biomass (Mantel’s r > 0.2, *P* < 0.05), SMC (Mantel’s r > 0.4, *P* < 0.01), NO_3_^−^-N (Mantel’s r > 0.4, *P* < 0.01), SOC (Mantel’s r > 0.2, *P* < 0.05), MBC (Mantel’s r > 0.4, *P* < 0.01), and MBN (Mantel’s r > 0.4, *P* < 0.01). The plant–parasite community was significantly positively correlated with MBN (Mantel’s r > 0.2, *P* < 0.05). The omnivore/predator community was strongly positively correlated with pH (Mantel’s r > 0.4, *P* < 0.05). The RDA also estimated the association between plant community, soil abiotic factors, and soil nematode community (**Figure 9B**; **Table S7**). Soil MBN (*r* = 0.69, *P* = 0.006) was the most important parameter contributing to the changes in soil nematode community, followed by soil MBC (*r* = 0.56, *P* = 0.027) and SMC (*r* = 0.61, *P* = 0.029). Meanwhile, SOC (*r* = 0.51, *P* = 0.038) and NO_3_^−^-N (*r* = 0.51, *P* = 0.043) also had significant impacts on nematode community. The above indicated that the changes in soil nematode community were mainly driven by soil C and N and SMC.

## 4 Discussion

Increasing cover crop diversity led to more plant community diversity in orchard (Gomez et al., 2018). High plant diversity could support multiple functions and sustain more ecosystem services by producing multiple substrates and changing habitat conditions (Spedding et al., 2004; Carrera et al., 2007; Isbell et al., 2011). In this study, we found that cover crop diversity greatly increased the plant diversity in orchard and also increased cover crop biomass and decreased weed biomass, and C8 treatment was better for controlling weed than C2 and C4 treatments. In addition, weed biomass was lower in C4 than that in C2 treatment. These may be because increasing plant diversity alters the composition of plant communities, reducing the richness of weed species (Norton et al., 2019). Furthermore, by selecting species with structural diversity in the distribution of biomass on the ground, multi-layered plant communities are formed to control the growth of weeds by cover crop growth, which competes with weeds for niche, resources, and light (Bjorn et al., 2019). On the basis of the findings observed by this study, which require further confirmation, it can be considered that, in the kiwifruit orchard among the different cover crop treatments, C8 treatment was the most effective in terms of weeds control to reduce the use of herbicides and preserve soil health as well as the sustainable development of the fruit industry.

Cover crop supported a higher nematode abundance than CK. This is likely because the cover crops supply more food resource for nematode. In this study, cover crop biomass was higher in cover crop treatments than that in CK and positively associated with total nematode abundance. Previous study found that higher plant species richness led to higher plant biomass, which support more abundance resources for nematode (DuPont et al., 2009). In this study, C4 and C8 increased SOC, TN, MBC, and MBN, indicating that cover crops enhance efficiency of soil resources, which further affects the soil nematode community, resulting in a higher abundance of soil nematodes under cover crop treatments than CK. In addition, cover crop diversity favored higher plant diversity, which increased substrates diversity that leads more diverse resources into soil and, subsequently, increases the richness and diversity of soil biota (Cesarz et al., 2017) Different cover crop treatments have different effects on the nematode trophic groups. Cover crop treatments supported a higher abundance of low trophic groups of free-living nematodes (bacterivores and fungivores), especially in C4 treatment. This may be related to the higher soil fertility and effective organic matter decomposition under cover crop diversity treatments (Housman et al., 2007; Wang et al., 2019). Mantel test revealed that MBC was positively correlated to bacterivores abundance. NO_3_^−^-N, SOC, MBC, and MBN were positively associated with fungivores abundance. Moreover, cover crops increased the biomass of bacterial and fungi (Novara et al., 2020) and may affect soil bacterivores and fungivores abundance through bottom-up control. Changes of soil environment can also affect nematode abundance. In this study, cover crop treatments increased SMC, which showed a positive correlation with fungivores abundance. This may be related to some fungivores, e.g., *Aphelenchoides*, which are more suitable for survival in environments with higher relative humidity (Zhao et al., 2022). Cover crop treatments impact plant parasites abundance (Yeates and Bongers, 1999; Verschoor et al., 2001), which is closely linked to the vigor of their host plants (Ferris et al., 2001) and benefited from high plant species diversity (Isbell et al., 2017). Cover crop treatments, especially C2 and C8, increased the abundance of plant parasites in this study. This is presumably due to the presence of root biomass, which provides abundant food resources for plant parasites nematodes. We also found that omnivore/predator nematode abundance was slightly increased in C8 treatment, implying that increasing cover crop diversity led to a more stable and less disturbed soil ecosystem in orchard, which provided an excellent living environment for omnivore/predator nematode. In addition to that, C8 increased the abundance of bacterivores, fungivores, and plant parasites, suggesting a strong bottom-up effect of cover crop on soil omnivore/predator nematodes (Hu et al., 2017). The soil nematode community composition was significantly affected by cover crop treatments. The reason for this outcome may be that different cover crop treatments have distinct effects on soil environmental factors because of their different identity, biomass, function, and root exudates, which, to some extent, alter soil properties (Pan et al., 2016). In this study, soil NO_3_^−^-N, MBN, MBC, SMC, and SOC were the important parameters contributing to the changes in soil nematode community composition. Previous study found that the dominant groups were *Rhabditis*, *Cephalobus persegnis*, and *Aporcelaimellus* in kiwifruit orchard soils (Sekhukhune et al., 2022a), whereas in our study, *Aphelenchoides*, *Eucephalobus*, and *Rhabditis* were the dominant genus. This difference may be caused by cover crops, which improved soil properties and increased the resource availability, subsequently affecting the composition of nematode communities (Zhang et al., 2022). The dominant groups of plant parasites (*Pratylenchus*, *Tylenchorhynchus*, and *Tylenchus*) in our study were also different from Sekhukhune et al. (2022b), who found that *Helicotylenchus*, *Meloidogyne hapla*, and *Scutellonema brachyurus* were the prevalent nematodes in kiwifruit orchard soil. This may be due to different soil management measures including fertilization, tillage, planting, and covering crops. Nematode network always varies when the composition of the soil nematode community has been changed. The number of nodes and edges networks can represent the size of ecological networks (Wu et al., 2021). In this study, the numbers of nodes and edges for nematode communities in cover crop soils were greater than those in CK soils, indicating that cover crops exhibit a larger network size and recruits more nematodes participating in the nematode–nematode interactions than those in CK. Cover crop also increased the density of nematode ecological networks, indicating that cover crop treatments enhanced the interaction between nematodes. The positive and negative correlations in the network can represent the collaboration and competitive predation relationships between nematodes, respectively (Wu et al., 2021). In this study, cover crop treatments reduced the positive correlation proportion and increased the proportion of negative correlation, suggesting that a strong competitive relationship between nematodes under the cover crop treatments. Compared with CK, the main genera changed in the network of cover crop treatments belonging to bacterivores (e.g., *Rhabditis*, *Protorhabditis*, *Caenorhabditis*, *Eumonhystera*, *Chiloplacus*, *Acrobeles*, and *Prochromadora*). These changes may be due to the fact that the cover crop treatments increased the resources input, which stimulates bacterivores to respond quickly. In addition, C2 and C8 increased the interactions between plant parasites (e.g., *Malenchus*, *Helicotylenchus*, and *Longidorus*) and other nematodes, probably related to the host plant and its root biomass and exudation. The network complexity of the feeding links was significantly correlated with the stability and stress tolerance of the soil food web (Beckerman et al., 2006; Wang et al., 2022), and our findings thus highlighted that cover crop diversity profited to the stability and interactions of the soil micro-food web.

The changes of soil nematode composition cause significant variations in nematode metabolic footprints. The functional metabolic footprint was higher in C4 and C8 compared with CK, indicating that cover crop diversity increased carbon utilization efficiency of soil nematode communities. This result is likely due to the fact that C4 and C8 increased the proportion of nematodes in the cp1 group. Soil nematode with low cp value was r-strategists, which respond quickly to external nutrient inputs, resulting in higher resource utilization rates (Zhao et al., 2022). In addition, the functional metabolic footprint in C8 was higher than that in C2 and C4, suggesting that C8 may have a stronger promoting effect on the metabolic activity of predatory omnivorous nematodes. The reason might be that C8 causes a larger cover crop biomass, which has a good nutrient enrichment status in the soil, and the structure of the soil food web is mature and stable. The bottom-up effect caused by resource input promotes the energy flow in the soil food web from low trophic level to high trophic level and maintains the metabolic activity of the soil food web. In this study, cover crop treatments also increased the metabolic footprint of fungivores, indicating that the carbon and energy flow entering the fungal decomposition pathway are relatively higher in the cover crop treatments than in CK. This result can be explained as cover crop treatments may be increase the refractory organic matters. In addition, C2 and C4 led to a higher fungivores metabolic footprint as compared with C8, possibly because C2 and C4 increased the proportion of fungivores compared with C8. In this study, cover crops also improved ecological index of nematode. C4 increased the *WI* but decreased the *PPI*, suggesting that the soil mineralization pathway under C4 treatment is mainly involved by bacterivorous and fungivores (Bongers, 1990). Meanwhile, C4 reduced the harm of plant parasitic nematodes in the soil food web and improved soil health. The changes of soil nematode composition led to changes of soil food web structure *via* influencing metabolic footprint. Soil nematode faunal analysis is based on nematode metabolic footprints, indicating that nutrient status is better, soil is less disturbed, and the food web is mature and stable within C8 soils compared with CK soils in the orchard ecosystem. This is likely due to the fact that C8 improved soil carbon and nitrogen content and created more enriched conditions (Ferris et al., 2001). Using soil nematodes as indicator taxa, our study shows that cover crops can maintain soil food web complexity and promote nutrients enrichment as well as the ecosystem stability, especially under C4 and C8 treatments.

## 5 Conclusion

Our findings demonstrated the large effect of increasing cover crop diversity on weed control and soil nematode community in kiwifruit orchard. Weed biomass presented lower in cover crop treatments and decreased as cover crop diversity increased. In addition, cover crop treatments increased the abundances of total nematode and fungivores, changed the structure of nematode community composition, and enhanced the complexity and interactions of the soil micro-food web. Moreover, C4 increased the *MI* but decreased the *PPI*. In addition, C4 and C8 increased the functional metabolic footprint of nematode. Thereby, this study screened C4 and C8 treatments, which were more effective in improving soil quality, nematode abundance, and soil food web, and provided theoretical basis for the scientific management of orchards, biodiversity conservation, and ecological restoration of degraded orchards.

## Data availability statement

The original contributions presented in the study are included in the article/**Supplementary Material**. Further inquiries can be directed to the corresponding authors.

## Author contributions

Conceptualization: DY and HW. Data curation: Q-ML. Formal analysis: Q-ML. Funding acquisition: DY and HW. Investigation: Q-ML, X-XQ, and HW. Methodology: DY, HW, J-NZ, and Q-ML. Project administration: DY and HW. Resources: H-ML, H-FZ, and Y-JZ. Software: Q-ML. Supervision: DY and HW. Validation: DY and HW. Writing—original draft: Q-ML. Writing—review and editing: Q-ML, DY, and HW. All authors contributed to the article and approved the submitted version.
