# Supplementary material for: Responses of soil nematode abundance and food web to cover crops in a kiwifruit orchard
Source: Front Plant Sci. 2023 Aug 3;14:1173157. doi: 10.3389/fpls.2023.1173157 (PMC10435974; doi:10.3389/fpls.2023.1173157)
Supplement: Supplementary file 1 [file Table_1.doc]

**Supplemental Information for**

Responses of Soil Nematode abundance and food web to Cover Crops in a Kiwifruit Orchard

**Table and figure of content**

**Table S1** Horticultural characteristics and seeding rates for eight cover crop species used in the field experiment.

**Table S2** Results (F and *P* values) of one-way ANOVAs of the effects of the cover crop treatments on plant, soil physiochemical factors, and nematode abundance ecological index and metabolic footprint.

**Table S3** Mean (n=3) abundance (individuals 100 g^−1^ dry soil) of nematodes functional guilds under different cover crop treatments.

**Table S4** The co - occurrence network topological properties of soil nematode community under different cover crop treatments.

**Table S5** The enrichment footprints (*Fe*), and structure footprints (*Fs*) of nematodes under different cover crop treatments.

**Table S6** The functional metabolic footprint is depicted by sequentially joining points under different cover crop treatments.

**Table S7** Conditional term effects according to the redundancy analysis between soil nematode community and plant, soil physicochemical factors.

**Table S1** Horticultural characteristics and seeding rates for eight cover crop species used in the field experiment.

| Common name | Scientific name | Height (cm) | Function | Cover crop seeding rate (g m^-2^) | | |
| --- | --- | --- | --- | --- | --- | --- |
|  |  |  |  | C2 | C4 | C8 |
| Ryegrass | *Lolium perenne* | 30-90 | Poaceae | 15 | 9 | 4.5 |
| Bluegrass | *Poa pratensis* | 10-30 |  |  | 12 | 6 |
| Red fescue | *Festuca rubra.* | 40-60 |  |  |  | 12 |
| White clover | *Trifolium repens* | 10-30 | Fabaceae | 10 | 6 | 3 |
| Red clover | *Trifolium pratense.* | 20-30 |  |  | 3.6 | 1.8 |
| Hairy vetch | *Vicia villosa* | 40-60 |  |  |  | 1.4 |
| Calliopsis | *Cosmos bipinnata* | 30-100 | Asteraceae |  |  | 1.8 |
| Zinnia | *Zinnia elegans* | 30-100 |  |  |  | 1.5 |

**Table S2** Results (F and *P* values) of one-way ANOVAs of the effects of the cover crop treatments on plant, soil physiochemical factors, and nematode abundance ecological index and metabolic footprint.

| Treatments | Df | F | *P* value |
| --- | --- | --- | --- |
| Plant |  |  |  |
| Cover crop biomass (g m^-2^) | 11 | 120.218 | < 0.001 |
| Weed biomass (g m^-2^) | 11 | 335.300 | < 0.001 |
| Shannon index (*H*) | 11 | 161.153 | < 0.001 |
| Soil physiochemical factors |  |  |  |
| SMC (%) | 11 | 8.397 | 0.007 |
| pH | 11 | 15.498 | 0.001 |
| NH_4_^+^-N (mg kg^-1^) | 11 | 3.109 | 0.089 |
| NO_3_^-^-N (mg kg^-1^) | 11 | 31.833 | <0.001 |
| TN (g kg^-1^) | 11 | 43.000 | <0.001 |
| SOC (g kg^-1^) | 11 | 21.947 | <0.001 |
| C/N | 11 | 14.722 | 0.001 |
| MBC (mg kg^-1^) | 11 | 31.111 | <0.001 |
| MBN (mg kg^-1^) | 11 | 24.764 | <0.001 |
| Nematode abundance |  |  |  |
| Total nematode abundance | 11 | 24.119 | 0.009 |
| Ba abundance | 11 | 15.116 | 0.121 |
| Fu abundance | 11 | 31.912 | 0.003 |
| Pp abundance | 11 | 5.687 | 0.003 |
| Op abundance | 11 | 13.160 | 0.255 |
| Nematode ecological index |  |  |  |
| *MI* | 11 | 1.790 | 0.227 |
| *PPI* | 11 | 3.160 | 0.086 |
| *NCR* | 11 | 0.076 | 0.971 |
| *WI* | 11 | 7.203 | 0.012 |
| Nematode metabolic footprint | | | |
| BaF | 11 | 2.391 | 0.144 |
| FuF | 11 | 12.693 | 0.002 |
| PpF | 11 | 2.449 | 0.138 |
| OpF | 11 | 0.655 | 0.602 |

Note: SMC, soil moisture content; NH_4_^+^-N, ammonium nitrogen; NO_3_^-^-N, nitrate nitrogen; TN, total nitrogen; SOC, soil organic carbon; C/N, carbon/nitrogen; MBC, microbial biomass carbon; MBN, microbial biomass nitrogen; Ba, Bacterivores; Fu, Fungivores; Pp, plant parasites; Op, Omnivores/predators; BaF, Bacterivores metabolic footprint; FuF, Fungivores metabolic footprint; PpF, plant parasites metabolic footprint; OpF, Omnivores/predators metabolic footprint.

**Table S3** Mean (n=3) abundance (individuals 100 g^−1^ dry soil) of nematodes functional guilds under different cover crop diversity treatments.

| Genus | c-p value | CK | C2 | C4 | C8 |
| --- | --- | --- | --- | --- | --- |
| **Bacterivorous** |  |  |  |  |  |
| *Mesorhabditis* | Ba1 | 21.23±14.43 | 109.58±66.22 | 134.31±54.32 | 111.67±24.30 |
| *Rhabditis* | Ba1 | 101.27±64.37 | 98.77±27.74 | 231.25±17.24 | 214.48±39.88 |
| *Pelodera* | Ba1 | 0.00±0.00 | 8.47±8.47 | 0.00±0.00 | 0.00±0.00 |
| *Protorhabditis* | Ba1 | 0.00±0.00 | 0.00±0.00 | 17.92±9.40 | 7.88±7.88 |
| *Ablechroiulus* | Ba1 | 5.42±5.42 | 6.98±6.98 | 0.00±0.00 | 0.00±0.00 |
| *Caenorhabditis* | Ba1 | 0.00±0.00 | 51.23±6.60 | 47.19±24.14 | 40.07±23.04 |
| *Diploscapter* | Ba1 | 16.26±16.26 | 0.00±0.00 | 0.00±0.00 | 0.00±0.00 |
| *Eucephalobus* | Ba2 | 134.62±56.70 | 246.22±110.69 | 277.71±36.15 | 133.19±18.62 |
| *Eumonhystera* | Ba2 | 15.90±0.50 | 22.12±1.67 | 69.88±13.75 | 30.22±5.16 |
| *Cephalobus* | Ba2 | 0.00±0.00 | 43.08±20.43 | 14.64±14.64 | 0.00±0.00 |
| *Chiloplacus* | Ba2 | 47.36±15.66 | 93.67±8.81 | 70.62±30.92 | 39.09±8.56 |
| *Plectus* | Ba2 | 59.27±20.69 | 74.20±26.63 | 93.31±2.76 | 113.98±34.35 |
| *Acrobeloides* | Ba2 | 138.25±13.87 | 119.41±21.17 | 150.76±21.30 | 126.79±36.81 |
| *Acrobeles* | Ba2 | 9.95±9.95 | 83.95±32.62 | 25.98±2.99 | 32.51±20.69 |
| *Anaplectus* | Ba2 | 0.00±0.00 | 16.95±16.95 | 0.00±0.00 | 8.87±8.87 |
| *Wilsonema* | Ba2 | 0.00±0.00 | 0.00±0.00 | 0.00±0.00 | 7.88±7.88 |
| *Prismatolaimus* | Ba2 | 140.38±52.94 | 59.06±19.09 | 83.78±37.68 | 211.19±29.37 |
| *Chromadorina* | Ba3 | 0.00±0.00 | 6.98±6.98 | 0.00±0.00 | 0.00±0.00 |
| *Prochromadora* | Ba3 | 5.51±5.51 | 0.00±0.00 | 0.00±0.00 | 24.47±15.51 |
| **Fungivorous** |  |  |  |  |  |
| *Aphelenchus* | Fu2 | 94.19±31.30 | 120.01±40.03 | 139.03±34.96 | 116.44±12.82 |
| *Ditylenchus* | Fu2 | 75.26±28.51 | 133.59±35.74 | 170.55±84.90 | 88.35±24.21 |
| *Aphelenchoides* | Fu2 | 209.95±38.96 | 334.96±56.19 | 416.73±94.34 | 443.57±73.29 |
| *Filenchus* | Fu2 | 57.57±12.57 | 114.23±34.38 | 190.25±47.88 | 151.74±44.51 |
| *Dorylaimoides* | Fu4 | 5.42±5.42 | 29.43±18.26 | 7.32±7.32 | 0.00±0.00 |
| *Tylencholaimus* | Fu4 | 5.51±5.51 | 15.46±7.84 | 0.00±0.00 | 0.00±0.00 |
| **Plant parasites** |  |  |  |  |  |
| *Tylenchus* | Pp2 | 0.00±0.00 | 37.90±19.26 | 35.85±18.81 | 96.24±34.01 |
| *Malenchus* | Pp2 | 36.88±10.40 | 15.46±7.84 | 60.75±21.61 | 31.37±8.18 |
| *Psilenchus* | Pp2 | 37.85±15.02 | 23.61±14.79 | 14.64±14.64 | 30.38±21.10 |
| *Tylenchorhynchus* | Pp3 | 36.78±4.00 | 55.77±22.61 | 14.64±14.64 | 32.35±10.47 |
| *Pratylenchus* | Pp3 | 20.79±10.42 | 70.91±24.67 | 62.56±16.50 | 96.23±29.73 |
| *Helicotylenchus* | Pp3 | 15.81±9.40 | 49.10±16.16 | 0.00±0.00 | 0.00±0.00 |
| *Rotylenchus* | Pp3 | 0.00±0.00 | 13.97±13.97 | 0.00±0.00 | 15.76±15.76 |
| *Longidorella* | Pp4 | 0.00±0.00 | 0.00±0.00 | 0.00±0.00 | 7.88±7.88 |
| *Longidorus* | Pp5 | 26.48±14.41 | 16.95±16.95 | 0.00±0.00 | 47.95±16.65 |
| **Omnivore-predator** |  |  |  |  |  |
| *Granonchulus* | Op4 | 0.00±0.00 | 0.00±0.00 | 10.61±10.61 | 0.00±0.00 |
| *Mylonchulus* | Op4 | 5.42±5.42 | 20.31±11.55 | 0.00±0.00 | 28.08±17.64 |
| *Anatonchus* | Op4 | 0.00±0.00 | 26.98±17.58 | 8.05±8.05 | 0.00±0.00 |
| *Clarkus* | Op4 | 0.00±0.00 | 8.47±8.47 | 0.00±0.00 | 24.63±13.69 |
| *Eudorylaimus* | Op4 | 48.78±19.55 | 61.51±23.59 | 64.04±16.71 | 114.14±14.06 |
| *Thonus* | Op4 | 68.94±14.59 | 6.98±6.98 | 88.88±68.11 | 65.69±34.01 |
| *Aporcelaimus* | Op5 | 20.88±4.49 | 13.65±6.83 | 17.92±9.40 | 34.48±23.67 |
| *Discolaimium* | Op5 | 0.00±0.00 | 8.47±8.47 | 0.00±0.00 | 0.00±0.00 |
| *Mesodorylaimus* | Op5 | 16.53±16.53 | 30.60±10.13 | 51.96±5.98 | 25.62±15.39 |
| *Sectonema* | Op5 | 4.98±4.98 | 0.00±0.00 | 0.00±0.00 | 8.87±8.87 |
| *Prodorylaimus* | Op5 | 81.66±49.67 | 0.00±0.00 | 26.71±14.18 | 15.60±8.02 |
| *Oxydirus* | Op5 | 35.27±27.55 | 15.14±7.73 | 36.58±13.54 | 31.37±8.18 |

Note: C2, two cover crop species; C4, four cover crop species; C8, eight cover crop species; CK, no cover crop. Values represents means of the three replications.

**Table S4** The co - occurrence network topological properties of soil nematode community under different cover crop treatments.

| Treatments | Ba nodes | Fu nodes | Pp nodes | Op nodes | Total nodes | Total edges | Degree | Network density |
| --- | --- | --- | --- | --- | --- | --- | --- | --- |
| CK | 12 | 6 | 6 | 8 | 32 | 86 | 5.375 | 0.173 |
| C2 | 15 | 6 | 8 | 9 | 38 | 167 | 8.789 | 0.238 |
| C4 | 12 | 5 | 5 | 8 | 30 | 91 | 6.067 | 0.209 |
| C8 | 14 | 4 | 8 | 9 | 35 | 135 | 7.714 | 0.227 |

Note: C2, two cover crop species; C4, four cover crop species; C8, eight cover crop species; CK, no cover crop.

**Table S5** The enrichment footprints (*Fe*), and structure footprints (*Fs*) of nematodes under different cover crop treatments.

| Treatments | *Fe* | *Fs* |
| --- | --- | --- |
| CK | 371.88±118.86b | 1155.63±437.56a |
| C2 | 534.98±107.13ab | 690.92±134.52a |
| C4 | 768.13±59.89a | 628.79±142.56a |
| C8 | 727.85±108.51a | 1630.36±714.16a |
| df | 11 | 11 |
| F | 3.283 | 1.175 |
| *P* | 0.079 | 0.378 |

Note: C2, two cover crop species; C4, four cover crop species; C8, eight cover crop species; CK, no cover crop. The different lowercase letter indicates significant differences among treatments according to Duncan's test (*P* < 0.05).

**Table S6** The functional metabolic footprint is depicted by sequentially joining points under different cover crop treatments.

| Treatments | (*SI*-0.5**Fs*/k, *EI*) | (*SI*, *EI*+0.5**Fe*/k) | (*SI*+0.5**Fs*/k, *EI*) | (*SI*, *EI*-0.5**Fe*/k) | (*SI*, *EI*) |
| --- | --- | --- | --- | --- | --- |
| CK | (45.13, 47.64) | (59.58, 52.29) | (74.02, 47.64) | (59.58, 43.00 | (59.58, 47.64) |
| C2 | (34.35, 54.30) | (42.99, 60.99) | (51.62, 54.30) | (42.99, 47.61) | (42.99, 54.30) |
| C4 | (38.49, 60.53) | (46.35, 70.13) | (54.21, 60.53) | (46.35, 50.93) | (46.35, 60.53) |
| C8 | (31.67, 59.77) | (52.05, 68.87) | (72.43, 59.77) | (52.05, 50.67) | (52.05, 59.77) |

Note: C2, two cover crop species; C4, four cover crop species; C8, eight cover crop species; CK, no cover crop. *EI*, enrichment index; *SI*, structure index; *Fe*, enrichment footprints; *Fs*, structure footprints. k, k is the conversion constant.

**Table S7** Conditional term effects according to the redundancy analysis between soil nematode community and plant, soil physicochemical factors.

| Environmental factors | RDA1 | RDA2 | ef.vectors.r | ef.vectors.pvals |
| --- | --- | --- | --- | --- |
| MBN | 0.97 | -0.23 | 0.69 | 0.006 |
| MBC | 0.92 | -0.39 | 0.56 | 0.027 |
| SMC | 0.92 | -0.40 | 0.61 | 0.029 |
| SOC | 0.91 | -0.41 | 0.51 | 0.038 |
| NO_3_^–^-N | 0.90 | -0.45 | 0.51 | 0.043 |
| Cover crop biomass | 0.90 | -0.43 | 0.41 | 0.087 |
| C/N | 0.48 | -0.88 | 0.38 | 0.114 |
| TN | 0.80 | 0.60 | 0.33 | 0.165 |
| Plant diversity | 0.74 | -0.68 | 0.24 | 0.297 |
| pH | 0.84 | -0.55 | 0.15 | 0.511 |
| NH_4_^+^-N | 0.90 | 0.44 | 0.14 | 0.526 |

Note: MBN, microbial biomass nitrogen; MBC, microbial biomass carbon; SMC, soil moisture content; SOC, soil organic carbon; NO_3_^–^-N, nitrate nitrogen; C/N, soil organic carbon/ total nitrogen; TN, total nitrogen; NH_4_^+^-N, ammonium nitrogen.
